# EMG-Spectrogram-Empowered CNN Stroke-Classifier Model Development

**DOI:** 10.3390/life16010114

**Published:** 2026-01-13

**Authors:** Katherine, Riries Rulaningtyas, Kalaivani Chellappan

**Affiliations:** 1 Biomedical Engineering, Department of Physics, Faculty of Science and Technology, Universitas Airlangga, Surabaya 60115, Indonesia; katherine.bme.research@gmail.com; 2 Department of Electrical, Electronics and System Engineering, Faculty of Engineering and Built Environment, Universiti Kebangsaan Malaysia, Bangi 43600, Malaysia; kckalai@ukm.edu.my

**Keywords:** EMG, deep learning, spectrogram, stroke classification, CNN

## Abstract

Stroke is a leading cause of death and long-term disability worldwide, with ischemic stroke accounting for approximately 62.4% of all cases. This condition often results in persistent motor dysfunction, significantly reducing patients’ productivity. The effectiveness of rehabilitation therapy is crucial for post-stroke motor recovery. However, limited access to rehabilitation services particularly in low- and middle-income countries remains a major barrier due to a shortage of experienced professionals. This challenge also affects home-based rehabilitation, an alternative to conventional therapy, which primarily relies on standard evaluation methods that are heavily dependent on expert interpretation. Electromyography (EMG) offers an objective and alternative approach to assessing muscle activity during stroke therapy in home environments. Recent advancements in deep learning (DL) have opened new avenues for automating the classification of EMG data, enabling differentiation between post-stroke patients and healthy individuals. This study introduces a novel methodology for transforming EMG signals into time–frequency representation (TFR) spectrograms, which serve as input for a convolutional neural network (CNN) model. The proposed Tri-CCNN model achieved the highest classification accuracy of 93.33%, outperforming both the Shallow CNN and the classic LeNet-5 architecture. Furthermore, an in-depth analysis of spectrogram amplitude distributions revealed distinct patterns in stroke patients, demonstrating the method’s potential for objective stroke assessment. These findings suggest that the proposed approach could serve as an effective tool for enhancing stroke classification and rehabilitation procedures, with significant implications for automating rehabilitation monitoring in home-based rehabilitation (HBR) settings.

## 1. Introduction

Stroke is a leading cause of mortality and long-term disability, particularly in low- and middle-income countries (LMICs) [1,2]. According to the Global Stroke Fact Sheet 2022, stroke accounts for 43% of total deaths and leads to a 143% loss in disability-adjusted life years (DALYs) worldwide. Globally, stroke is the second leading cause of death and the third leading cause of disability globally [2,3]. The incidence of stroke has increased by approximately 70%, resulting in a significant economic burden exceeding $721 billion annually. Notably, LMICs account for 86% of all stroke-related deaths and 89% of the total DALY losses, and the risk of stroke is expected to increase by 50% in the coming decades [1]. Furthermore, the prevalence of stroke in individuals aged less than 70 years among individuals under 70 has increased by 22%, underscoring the increasing need for effective rehabilitation strategies [2].

Stroke occurs when the blood supply to the brain is interrupted, leading to two primary types: hemorrhagic and ischemic [4]. Among these, ischemic stroke accounts for 62.4% of all stroke events, affecting approximately 795,000 people annually, with one case occurring every 40 s [5,6,7]. The impact of stroke on survivors, particularly those affected by ischemic stroke, is profound, and often results in severe long-term motor impairment, which can greatly restrict mobility and independence [4]. This underscores the critical need for intensive rehabilitation to optimize recovery. However, access to rehabilitation services remains a challenge, with over 30% of stroke patients not receiving post-acute rehabilitation within the first 30 days after discharge [8]. Rehabilitation typically requires frequent therapy sessions, yet the limited availability of specialists has been identified as a key factor contributing to the low intensity of rehabilitation per week [9,10].

Although the pandemic has introduced new challenges, it has also accelerated the adoption of remote rehabilitation approaches, particularly home-based rehabilitation (HBR) models, which have gained growing recognition as viable alternatives for improving rehabilitation service accessibility [4,11]. However, a major limitation of HBR is the lack of real-time monitoring by healthcare professionals, which may lead to inadequate rehabilitation doses or excessive muscle effort, potentially resulting in fatigue or injury [12]. Currently, post-stroke muscle activity is primarily assessed through manual evaluation methods, such as the National Institutes of Health Stroke Scale (NIHSS), manual muscle testing (MMT), Fugl–Meyer Assessment (FMA), and range of motion (ROM) measurements [13,14,15,16,17]. These techniques require expert interpretation and may be impractical for unsupervised HBR patients [18]. Therefore, integrating a monitoring system into the HBR is crucial for objectively assessing motor recovery in stroke patients.

Recently biosignals, including electromyograms (EMGs), electroencephalograms (EEGs), electrocardiograms (ECGs), and photoplethysmogram (PPG), have been extensively used as monitoring systems [19,20,21,22,23]. Among these, EMG is the most widely employed signal for analyzing muscle biopotentials, which are crucial for assessing the rehabilitation progress. Electromyographic (EMG) signals recorded using surface electrodes placed on the muscle provide biofeedback on neuromuscular activity and contraction intensity [24,25,26,27,28]. The amplitude of an EMG signal correlates with the muscle contraction force, making it a reliable indicator of muscle function [29]. Unlike conventional manual assessments, EMG-based evaluation offers objective, real-time muscle activity monitoring, making it an ideal modality for post-stroke rehabilitation applications, particularly in HBR settings [21,24].

Recent studies have explored the potential of EMG signals for stroke rehabilitation monitoring and have demonstrated their effectiveness in home therapy monitoring systems. Combining EMG with kinematic sensors has achieved classification accuracies ranging from 80% to 96.43% for hand movement pattern recognition [30,31]. Additionally, some studies have directly classified EMG signals using Random Forest and Linear Regression models, reporting accuracies of 90.38% and 69.5%, respectively [32,33]. Furthermore, the application of machine-learning (ML) models such as Support Vector Machines (SVMs) has been explored for multi-channel EMG signal classification, achieving accuracies between 82% and 96.62% [34,35]. These findings underscore the potential of EMG-based classification for muscle assessment.

Deep learning (DL) has become a widely adopted approach for EMG signal classification, offering a transformative framework that enables automated feature extraction and classification. This method enhances neural network tuning, improves prediction performance, and addresses the limitations of traditional machine learning (ML), which relies heavily on expert-driven feature selection, combination, and extraction [36]. A notable study explored muscle contraction identification using multi-channel EMG signals and the K-Nearest Neighbors (KNN) algorithm, achieving a classification accuracy exceeding 90% [37]. Additionally, research has focused on differentiating stroke patients from healthy individuals. For instance, convolutional neural networks (CNNs) have been employed to classify biosignals, yielding accuracy rates between 70% and 86.66% [38,39]. In a separate study, the LSTM-Attention model, which incorporates memory units, demonstrated an accuracy of 91.7% [40]. Furthermore, a hybrid model combining CNN and LSTM methods achieved an accuracy of 72.95% [36]. This study also compared a one-dimensional EMG approach, which achieved only 68.38% accuracy, with a two-dimensional frequency characteristics approach, which yielded superior accuracy. These findings offer new insights into the potential of EMG in classification applications [36]. Additionally, a study that utilized power spectral density (PSD) highlighted the critical role of time–frequency domain spectrogram data in EMG signal evaluation [41].

In recent years, the demand for scalable and remote rehabilitation tools has increased, particularly considering resource limitations and the growing role of telemedicine. While traditional stroke rehabilitation relies heavily on in-person assessments of motor function, there is a critical gap in the development of objective home-compatible tools for evaluating neuromuscular recovery. The ability to classify EMG signals from stroke patients versus healthy individuals can support the remote monitoring of muscle reactivation and neuromuscular improvement [42]. By embedding this classification into a tele-rehabilitation framework, clinicians can detect early signs of deterioration or improvement and guide timely adjustments in therapy without requiring physical clinic visits. This study aimed to lay the groundwork for intelligent remote monitoring systems by developing a CNN-based model trained on spectrogram-transformed EMG data to distinguish post-stroke from control muscle activity. A CNN was adopted as the primary deep learning architecture because of its well-established efficacy in handling two-dimensional image classification tasks [43].

## 2. Materials and Methods

### 2.1. Data Collection

This study recruited a cohort of healthy controls and post-stroke patients to examine hand muscle activity during grasping movements. Data were acquired from the Universitas Airlangga Hospital and the Biomedical Engineering Laboratory. The focus on the hand muscles is due to their essential impairment in post-stroke individuals.

#### 2.1.1. Subjects

The study involved 20 volunteers, and data acquisition was conducted at the Universitas Airlangga Hospital and Biomedical Engineering Laboratory. Ten healthy volunteers (5 males and 5 females, aged 21–50 years) participated as control subjects, they had no history of stroke, no familial relationship with stroke patients and no muscle-related disorders. Additionally, ten post-stroke patients (five males and five females, aged 37–71 years) were included in the study. The inclusion criteria required patients to have experienced an ischemic stroke; exhibit one-sided paralysis of the upper extremities, particularly affecting the fingers and hands; experience difficulty bending their fingers; and have MMT levels ranging from 0–4. Patients were excluded if they had finger contractures, structural abnormalities of the hand and fingers, unstable fractures, ideomotor apraxia, or severe cognitive impairments.

#### 2.1.2. Muscle Selection

The assessment of stroke patients is primarily based on the subjective evaluation of their ability to perform activities of daily living (ADLs). Post-stroke patients often experience a decline in ADL performance owing to motor impairment in the upper and lower extremities. This study focused on evaluating the upper extremities, specifically the hand and wrist, which play crucial roles in daily activities. Grasping, pressing, and touching are among the most frequently used moments of the hands and wrists, and are essential for performing everyday tasks. These movements are also the most frequently assessed movements within the grasping category, as highlighted by the Functional Independence Measure (FIM) stroke assessment. The FIM evaluates the post-stroke patients’ ability to perform essential ADLs, such as eating, drinking, grooming, bathing, and dressing tasks that rely heavily on grasping-related movements. Therefore, grasping was identified as a key motor function of the hand muscles and was the primary focus of this study.

**Figure 1:**
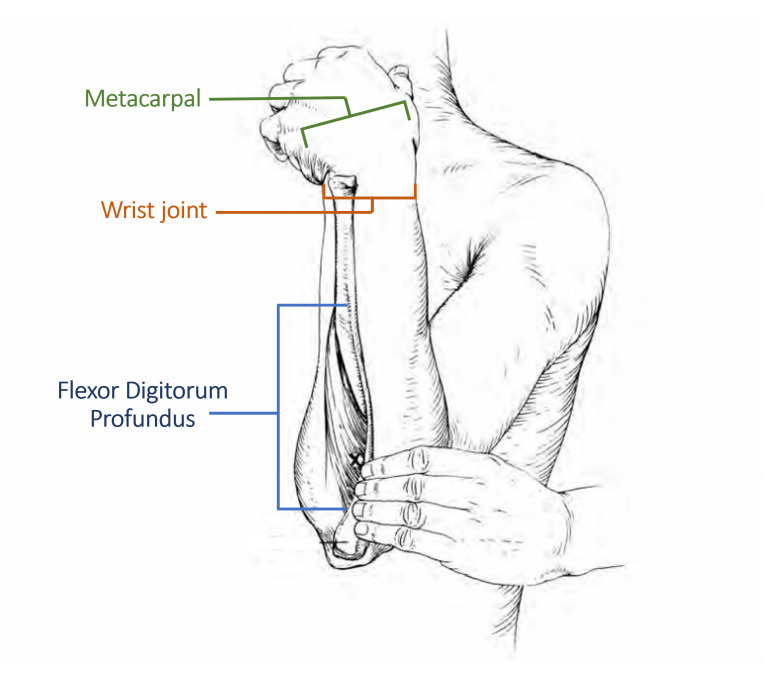
Flexor Digitorum muscle illustration [46].

**Figure 2:**
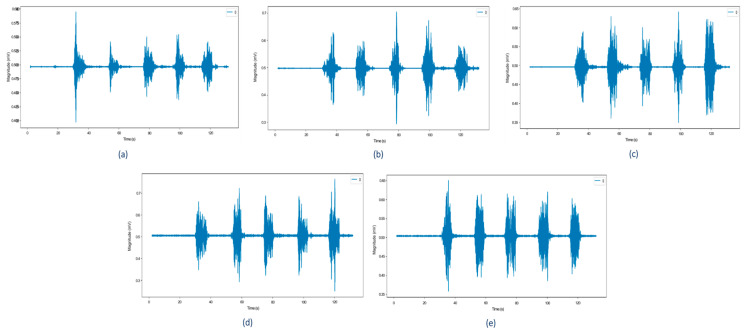
EMG signals of 5 control subjects, subject (**a**) 1, (**b**) 2, (**c**) 3, (**d**) 4, and (**e**) 5.

A literature review was conducted to identify the muscles that contributed the most to grasping movements. Based on their anatomical structure, grasping movements involve several muscles including the flexor pollicis brevis, extensor digitorum, extensor carpi radialis, flexor digitorum, flexor carpi ulnaris, and extensor carpi ulnaris [44,45,46]. In terms of strength, the extrinsic flexor muscle, the flexor digitorum profundus, contributes the most to grip strength [47]. The flexor digitorum is the only muscle that can flex the distal interphalangeal joints of all fingers, metacarpal-phalangeal joints, and the wrist joint, as shown in Figure 1 [46]. The contraction of the flexor digitorum profundus muscle provides electrical signals prior to the occurrence of movement, thus enabling the potential for more significant differences in biopotential activity to be read by EMG sensors using electrodes placed on the muscle belly [48]. Based on the literature, the flexor digitorum profundus muscle activity was recorded using EMG in five control subjects in a laboratory setting. The recorded data showed similar EMG patterns for all five subjects, as shown in Figure 2. Referring to the laboratory experiment outcome, the flexor digitorum profundus muscle activity was used to evaluate the grasping movement improvement classification in patients undergoing post-stroke rehabilitation.

#### 2.1.3. Experimental Protocol

**Figure 3:**
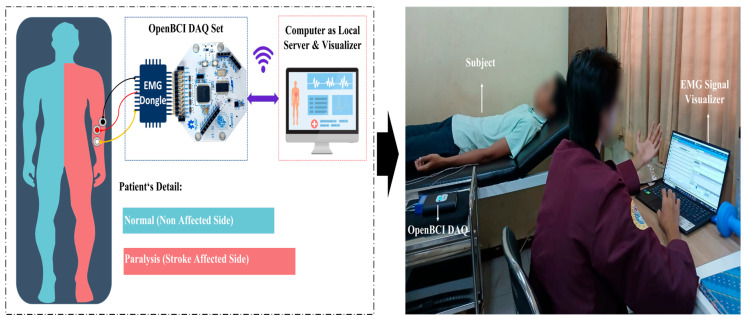
Experimental Setup.

**Figure 4:**
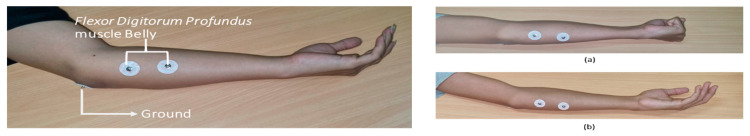
Electrode placement in Flexor Digitorum muscle, with (**a**) grasp movement, and (**b**) relaxation.

Each participant provided informed consent before the experimental procedure. The confidentiality of the subject’s personal data was assured, and the possibility of withdrawing from the study at any point throughout the experiment session was made clear to participants. The experimental protocol was approved by the Airlangga University Hospital Ethics Committee, Surabaya, Indonesia, (ethical approval number 125/KEP/2023) for data acquisition protocol. The data acquisition protocol for both control patients was identical, as listed in Table 1, and the experimental setup is shown in Figure 3. Table 1 shows that the subjects were positioned in the supine position with their entire bodies at rest, as shown in Figure 3. Before placing the electrode on the hand, the skin must be cleaned to ensure secure attachment and to prevent detachment during data acquisition [31]. To record the EMG signals, three electrodes were placed on the affected hand of post-stroke patients and on the left hand of control subjects. Small surface electrodes were used in this study to minimize the influence of the surface electric potential on the morphology of the EMG signals [49]. Two electrodes were placed on the muscle belly of the flexor digitorum profundus, the primary activity site, in a bipolar configuration to ensure precise time-delay measurements [50]. The third electrode was placed on the elbow as the ground reference, as shown in Figure 4. The EMG signals were recorded using an OpenBCI DAQ device (Meegi Air, Jakarta, Indonesia). For real-time monitoring, the device filter was set to 50–60 Hz, at a sampling rate of 250 Hz. Prior to the performance of the movement, a 30 s interval was allocated to ensure signal stability. The participants were instructed to perform a series of grasp movements, repeated five times consecutively, with rest intervals in between to prevent muscle fatigue, as shown in Figure 4. Each experimental session lasted 135 s, with each movement performed for a 6 s, followed by 15 s rest interval between movements.

### 2.2. Dataset Preparation

Raw EMG signals are often affected by noise from powerline interference, either from the device itself or from the surrounding environment. In addition, movement artifacts can cause signal distortion, necessitating robust pre-processing techniques to ensure clean signals before further analysis.

#### 2.2.1. Pre-Processing

The pre-processing procedure involved noise reduction using a 50 Hz notch filter to suppress powerline interference, following established EMG filtering practices [51]. This was followed by a fourth-order Butterworth band-pass filter (115–120 Hz) to eliminate motion artifacts and other unwanted noise [48]. Although EMG signals typically span 20–500 Hz, our preliminary spectral analysis revealed a consistent energy peak near 118 Hz, while lower frequencies were dominated by motion artifacts and higher frequencies by sensor noise. A Butterworth filter was chosen due to its stable passband characteristics and favorable roll-off performance compared with other commonly used filters [52,53]. The filtered EMG signals were subsequently normalized using Min–Max scaling to a 0–1 range. This normalization step ensured consistent amplitude scaling across subjects while preserving signal integrity and waveform characteristics.

#### 2.2.2. Segmentation

The normalized EMG signal is divided into segments to facilitate feature extraction. A disjoint-window technique, which is known for its low computational complexity, was employed for segmentation. To ensure consistency across all participants, the length of each EMG contraction signal window was fixed between 5 and 6 s. Following segmentation, each EMG contraction segment was labeled in accordance with the dataset structure listed in Table 2 for further processing.

#### 2.2.3. Time–Frequency Representation (TFR)

**Figure 5:**
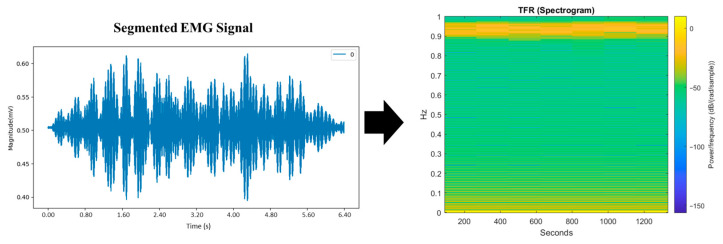
Transforming one-dimensional time signal (Segmented EMG Signal) into a two-dimensional TFR (Spectrogram).

One of the biopotential signals generated by the human body is the EMG signal, which falls under the category of biomedical signals and is characterized by its non-linear and non-stationary nature [54]. Consequently, analyzing EMG signals is a significant challenge. A powerful method for extracting deeper insights from these signals is to transform them into time–frequency (T-F) spectral images. This approach is applied to segmented EMG contraction data, which are then converted into a time–frequency representation (TFR) using Equation (1) [55]. Spectral characteristics are visualized as an energy distribution in a TFR or (spectrogram), capturing variations in frequency and amplitude over time. Leveraging the properties of the wavelet transform (WT) and short-time Fourier transform (STFT), the TFR provides a comprehensive representation of signals in both the time and frequency domains. This transformation converts a one-dimensional time signal into a two-dimensional time–frequency function, *Sb*(*τ*,*f*) as shown in Figure 5. Here, f1 represents the frequency, t denotes time and τ serves as the Gaussian window control parameter, which is used in the calculation and subsequently applied to Equation (1).

(1) $$S_{b}(\tau ,f)=\frac{\left|f_{1}\right|}{\sqrt{2\pi }}\int_{-\infty }^{\infty }b\left(t\right)e^{\frac{-{\left(\tau -t\right)}^{2}{f_{1}}^{2}}{2}}e^{-j2\pi f_{1,t}}dt$$

### 2.3. Data Classification

The TFR or spectrogram was used to convert the original one-dimensional EMG signal into a two-dimensional image, enabling classification into two categories: healthy subjects and stroke patients. A Convolutional Neural Network (CNN), a type of Deep Neural Network, was selected as the classifier for the spectrogram data. CNNs are an advanced development of the Multilayer Perceptron (MLP) specifically designed to process two-dimensional data [56]. They can automatically identify and categorize objects within an image by learning patterns directly from the data, thereby eliminating the need for manual feature extraction. To evaluate the effectiveness of the CNN in classifying spectrogram data, three distinct architectures were tested: Shallow CNN [56], classic LeNet-5 [57,58], and the proposed architecture named Tri-CCNN, which consists of three convolutional layers. The input images for the CNN were formatted to dimensions of 451 × 451 × 3, with identical hyperparameters applied across all three models to ensure a fair performance evaluation, as listed in Table 3.

The hyperparameters for the Tri-CCNN model were selected through an iterative tuning procedure combining manual exploration and constrained grid search. Initial ranges for learning rate (1 × 10^−4^ to 1 × 10^−2^), batch size (8–64), and epoch count (50–200) were evaluated to balance convergence stability and capacity to generalize on a limited dataset. Kernel and filter sizes across convolutional layers were progressively reduced to preserve salient EMG time–frequency features while minimizing information loss. The final configuration learning rate of 0.0001, batch size of 4, and 10 training epochs was selected based on the highest validation accuracy and lowest validation loss across three randomized dataset splits. No regularization beyond inherent architectural constraints was applied, as excessive regularization degraded performance due to the small dataset size. All experiments were executed using fixed random seeds to ensure reproducibility. The dataset was split into training and testing sets at three ratios: 70:30, 80:20, and 90:10.

**Training Techniques and Stopping Criteria**

All models were trained for a fixed number of 10 epochs with no early stopping or learning-rate scheduling. A constant learning rate of 0.0001 was used throughout training. Training was terminated strictly after completing the predefined epochs, as validation-based adaptive stopping was not applied. No additional regularization techniques (dropout, L1/L2 weight penalties, or batch normalization) were used, as preliminary experiments indicated reduced performance when applied to this small dataset. This training procedure ensures repeatability and mirrors the same stopping strategy across all three architectures for fair benchmarking.

A batch size of 4 and a learning rate of 0.0001 were selected to ensure stable training given the limited dataset of 100 segmented EMG samples. Smaller batch sizes are known to improve gradient estimation when data volume is restricted, reducing the risk of overfitting and allowing the model to capture subtle variations in EMG time–frequency patterns. Similarly, a small learning rate was chosen to maintain stable convergence and prevent oscillatory behavior during weight updates. Preliminary trials using larger batch sizes or higher learning rates resulted in inconsistent convergence and reduced classification performance. The selected configuration provided the most stable training dynamics across all three CNN architectures.

#### 2.3.1. Basic Architecture of CNN

A Convolutional Neural Network (CNN) consists of multiple layers, each of which is responsible for learning specific patterns to detect different features within an image. The CNN architecture includes an input layer, an output layer, and several hidden layers. The key component of a CNN is the convolutional layer that applies a convolution operation to extract features from the input image. This process involves repeatedly applying a kernel to the image at all possible offsets, thereby enabling the network to capture spatial patterns. Convolution facilitates the linear transformation of input data while preserving its spatial structure, making it a powerful technique for feature extraction. The convolution calculation process is expressed as follows Equation (2):

(2) f(i,j)=AP1+BP2+CP3+DP4+EP5+FP6+GP7+HP8+IP9

**Figure 6:**
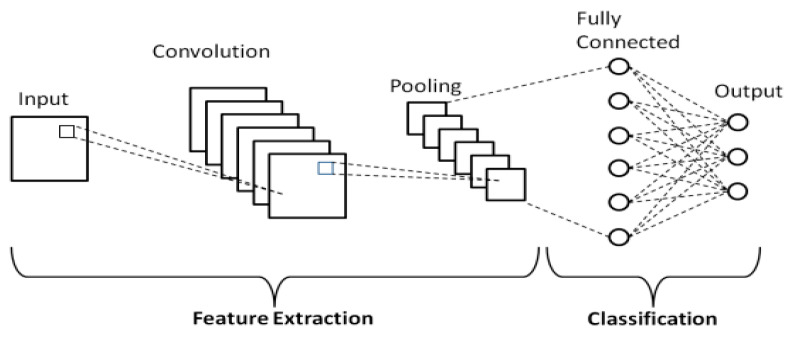
Basic architecture of Shallow CNN [47].

The basic CNN architecture comprises a single convolution layer and is regarded as the fundamental architectural construct, named a Shallow CNN, as shown in Figure 6 [56,59,60].

#### 2.3.2. Proposed Architecture: Tri-CCNN

**Figure 7:**
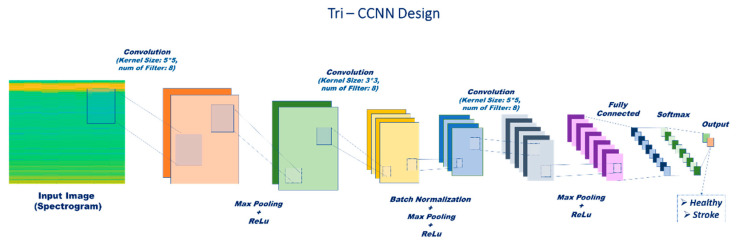
Architecture of Tri-CCNN.

A new convolutional neural network (CNN) architecture, Tri-CCNN, was introduced in this study as the core component of the proposed framework. The Tri-CCNN architecture was specifically designed to address the challenges inherent in EMG signal classification, particularly those related to small dataset sizes, non-stationary signal behaviors, and the need for localized feature extraction. Unlike standard deep CNNs, which risk overfitting due to large kernel and filter sizes, Tri-CCNN utilizes a progressive reduction in kernel dimensions across convolutional layers, allowing it to preserve essential local frequency–power features while controlling model complexity. This hierarchical refinement is well suited for EMG spectrograms, where motor unit activation patterns are often confined to narrow frequency bands. In addition, the network structure was optimized for generalization across multiple EMG feature domains, including spectrograms, amplitude spectra, and peak strength maps, without architectural changes. This flexibility underscores its value in diverse rehabilitation applications, particularly those relying on wearable or remote EMG monitoring. Tri-CCNN comprises three primary convolutional layers, as shown in Figure 7. Each convolutional layer utilizes a distinct kernel size, following an inverted triangular model with kernel sizes of 5 × 5, 3 × 3, and 2 × 2. After each convolutional layer, a max-pooling function with the same stride was applied, followed by a Rectified Linear Unit (ReLU) activation function [61]. To improve the stability of the feature recognition, Batch Normalization was incorporated after the second convolutional layer. Finally, a fully connected layer and Softmax activation function were used in the final classification layer. The filter parameters, strided max-pooling functions, and kernel sizes are listed in Table 4.

### 2.4. Performance Evaluation

The classification algorithm was evaluated using a standard confusion matrix to ascertain its accuracy [62,63]. In addition to accuracy, the specificity and sensitivity of each dataset and classifier were assessed.

1. Accuracy: Classification accuracy represents the extent to which instances are correctly classified as a proportion of the total number of instances. Equation (3) expresses the accuracy of the percentage term:

(3) $$Accuracy=\frac{\left(TP+TN\right)}{\left(TN+TP+FP+FN\right)}\times 100\%$$

2. Precision: Precision is defined as the ratio of positive predictive values to positive outcomes, where Equation (4) represents precision:

(4) $$\mathrm{Pr}ecision=\frac{TP}{\left(TP+FP\right)}\times 100\%$$

3. Sensitivity/Recall: Recall refers to the ratio of the true positive values to the total positive data, as represented by Equation (5).

(5) Recall=TPTP+FN×100%

4. F1-Score: The F1 score is determined as a weighted average of precision and recall, as represented by Equation (6).

(6) F1−Score=2×Precision×RecallPrecision+Recall×100%

## 3. Results

### 3.1. Data Descriptive Analysis

Twenty participants, comprising ten healthy controls (50%) and ten post-stroke patients (50%), were screened and enrolled in this study, as listed in Table 5. To consider the inherent variability and complexity of the EMG signals, control participants were selected to include a diverse range of ages and genders, with an equal distribution of five males and five females in each group. The age range of the control group was 21 to 50 years, with a mean age of 30 ± 8.65 years, whereas the post-stroke patient group had a mean age of 54 ± 10.93 years, ranging from 37 to 71 years. The post-stroke patient group consisted of individuals who had experienced ischemic stroke, with one participant (10%) having a family history of stroke. Notably, the dataset included both paralyzed patients (MMT 0) and those who had achieved maximal functional recovery within the stroke cohort (MMT 4) [14,16], as listed in Table 5. The control group comprised individuals with no family history of stroke, hand muscle anomalies, or congenital motor system disabilities.

The EMG signals obtained from each subject were recorded as raw numerical time-series data. These signals subsequently underwent a structured preprocessing pipeline that included initial filtering, signal normalization, and segmentation to extract individual EMG segments corresponding to each grasp contraction. Each subject performed five grasp cycles, yielding five contraction segments per participant and resulting in 50 data points for each group (control and post-stroke).

**Figure 8:**
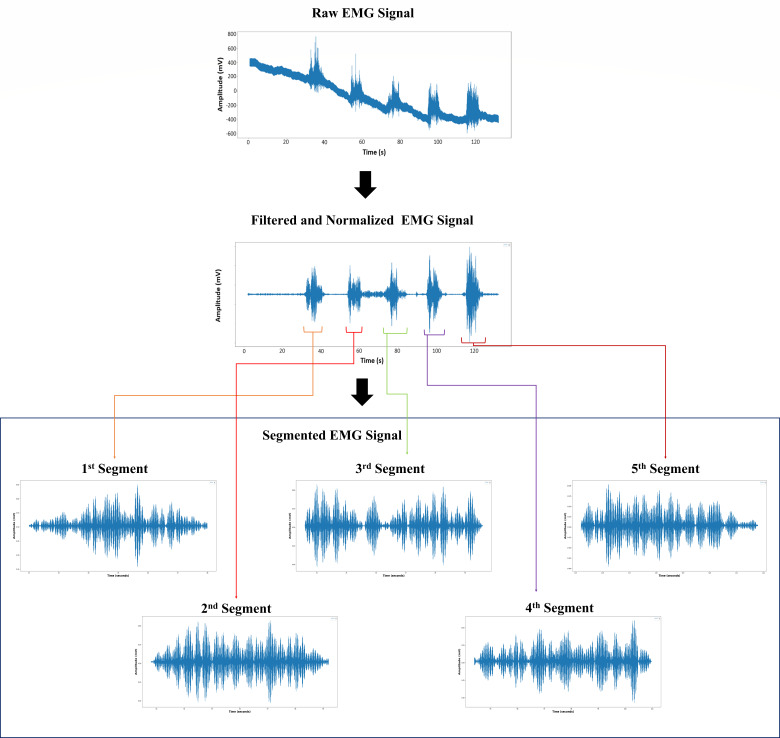
EMG preprocessing pipeline. (**Top**) Raw EMG signal showing five consecutive grasp cycles. (**Middle**) Filtered and normalized EMG signal containing the same five cycles. (**Bottom**) Five representative contraction segment extracted from the normalized signal, illustrating the fixed-length window used for spectrogram generation. All remaining grasp cycles undergo identical segmentation.

The EMG signals obtained from each subject are represented as raw signals in the form of numerical data. These signals then undergo preprocessing, including initial filtering and normalization of the EMG signals, followed by segmentation to extract an EMG segment for each contraction. This process resulted in five contraction segments per subject, providing 50 data points for each group (control and post-stroke patients). The outcome of each stage of preprocessing the raw EMG signal data is shown in Figure 8. The preprocessing process generates a total of 100 segmented EMG segments. These segments were labelled and converted into time–frequency representations as spectrogram images to form a dataset for further analysis.

The continuous EMG recording contains five voluntary grasp contractions per subject. After filtering and normalization, each contraction is segmented using a fixed time-based window. For visualization purposes, only the first contraction segment is shown in Figure 8 (top panel); however, all five contractions are segmented using the same procedure and included in the dataset. The bottom panel therefore represents a representative contraction segment rather than a separate recording.

### 3.2. Features Extraction and Image Generator

**Figure 9:**
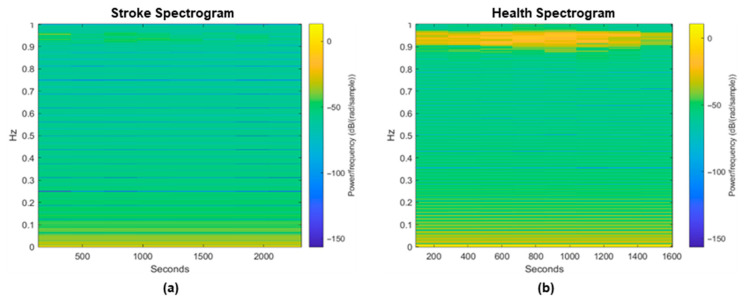
Spectrogram image for (**a**) post-stroke patient—P001 and (**b**) control subject—H001.

**Figure 10:**
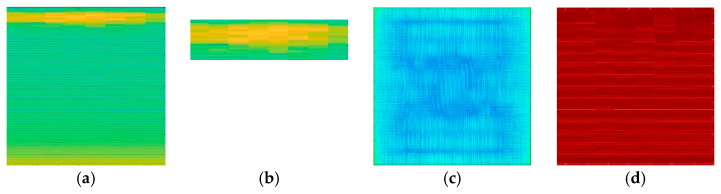
Four different datasets derived from the segmented EMG dataset, which is (**a**) Spectrogram, (**b**) Spectrogram Yellow ROI, (**c**) Amplitude Spectrum, and (**d**) Peak Strength.

Figure 9 shows the spectrograms from both the post-stroke and control groups, where a noticeable contrast in brightness levels is evident between the two groups. This difference reflects the power/frequency in dB/(rad/sample), particularly within the frequency range of 0.9–1 Hz. The spectrogram image was then further segmented at the frequency range of 0.9–1 Hz to create a second dataset for comparison, termed the Spectrogram Yellow Region of Interest (ROI), based on the visually identified ‘yellow line’ that appeared at this frequency in the spectrogram image. In addition, this study developed a two-dimensional visualization of the amplitude spectrum [64,65,66,67] and peak strength [68,69], providing two comparative datasets derived from previous studies. Figure 10 shows a qualitative comparison of the four image datasets.

The raw EMG data were processed in two steps. The first step is preprocessing, which involves filtering, normalizing, and segmenting each contraction. In the second step, the time-domain dataset is converted into the time–frequency domain and represented as a spectrogram. This spectrogram was then analyzed to extract three distinct datasets: the spectrogram yellow ROI, amplitude spectrum, and peak strength. These three datasets, along with the original spectrogram dataset, were used for modeling.

### 3.3. CNN Model Development and Classification Performance

In this study, three distinct CNN architectural models are trained using four different datasets. Training data were randomly selected using the system. The designed model, Tri-CCNN, optimizes the parameters in each convolution layer by adjusting the input data generated from the previous layer. The reduction in the input size produced by the previous convolution layer was accompanied by a decrease in the parameters, specifically the kernel and filter sizes. This approach enables optimization of the learned area while preserving the resulting features.

**Figure 11:**
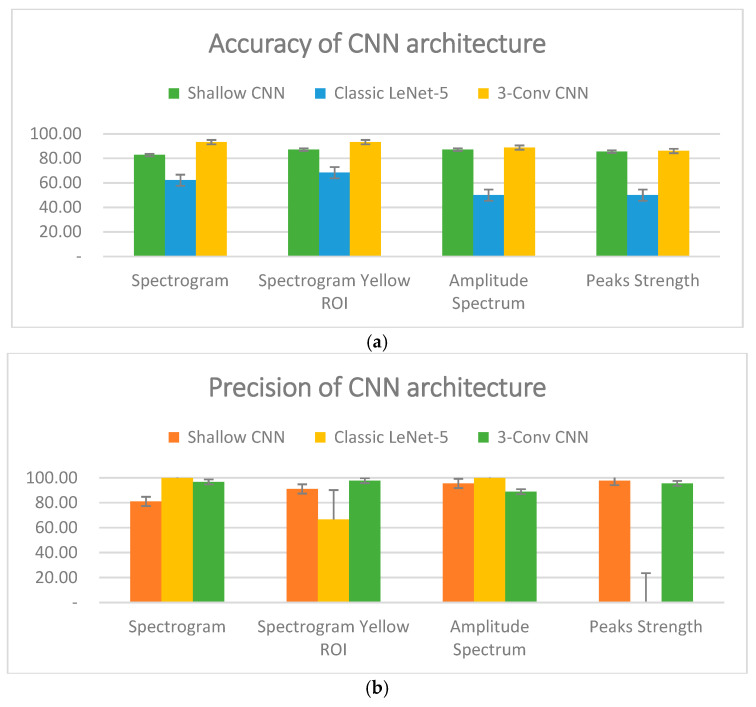
The average performance of all three CNN models for the entire dataset, (**a**) CNN accuracy and (**b**) CNN precision.

Across all datasets, the Tri-CCNN model demonstrated the highest overall performance based on the mean accuracy across the 70:30, 80:20, and 90:10 splits. As shown in Figure 11a, the Tri-CCNN model achieved mean accuracies of 93.33% for both the spectrogram and spectrogram yellow ROI datasets (95% CI: 86.7–96.8%), 88.87% for the amplitude spectrum (95% CI: 81.2–93.6%), and 86.10% for the peak-strength dataset (95% CI: 78.0–91.6%). The Shallow CNN yielded moderate yet consistent performance, with mean accuracies of 82.77% (95% CI: 74.2–88.9%) for the spectrogram dataset, 87.20% (95% CI: 79.3–92.4%) for both the spectrogram yellow ROI and amplitude spectrum datasets, and 85.53% (95% CI: 77.3–91.1%) for the peak-strength dataset. In contrast, the Classic LeNet-5 exhibited substantially lower performance, achieving mean accuracies of 62.20% (95% CI: 52.4–71.1%) for the spectrogram dataset, 68.33% (95% CI: 58.7–76.6%) for the spectrogram yellow ROI dataset, and only 50.00% (95% CI: 40.4–59.6%) for both the amplitude spectrum and peak-strength datasets. Overall, these results show that Tri-CCNN generalizes more effectively across input representations and data-split conditions compared with the other two architectures.

Table 6 lists a comparative benchmark of both the performance metrics and architectural design, allowing for an evaluation of the relative strengths of the proposed Tri-CCNN. The proposed model demonstrated the highest accuracy when using a spectrogram-based input, benefiting from the domain-specific kernel design and architectural tuning.

**Figure 12:**
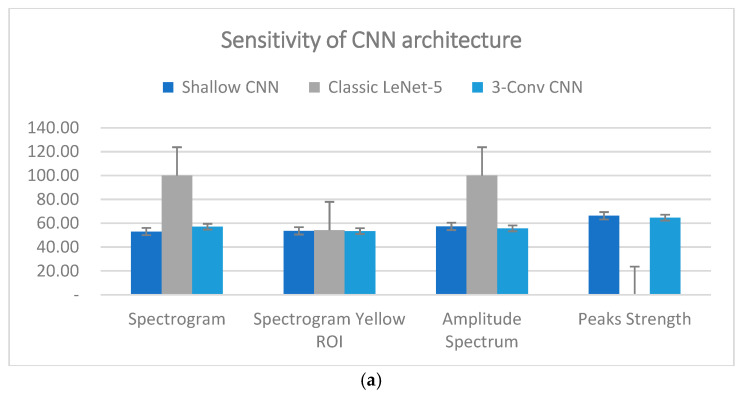

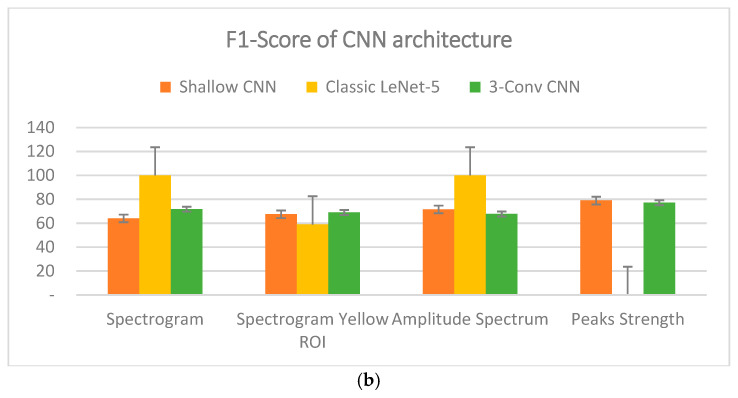
(**a**) sensitivity and (**b**) F1-Score classification performances.

**Figure 13:**
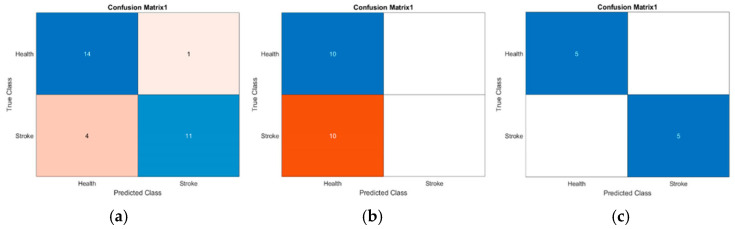
Confusion matrix result for spectrogram data using (**a**) shallow CNN (70:30), (**b**) classic LeNet 5 (80:20), and (**c**) Tri-CCNN (90:10).

Conversely, the performance of the Tri-CCNN model as a classifier showed no significant differences compared with the Shallow CNN and Classic LeNet-5 models (excluding the case of 0% or 100%) in terms of sensitivity and F1-score, as shown in Figure 12a,b. The Tri-CCNN model demonstrated a sensitivity range of 53.43–64.73%, whereas the Shallow CNN exhibited a slightly broader range of 53.03–66.30%. The sensitivity remains below 70%, indicating that the proportion of accurately identified negatives continues to contribute to higher prediction errors [54]. This is also reflected in the F1-score, which shows a decline in performance when the precision is high but the sensitivity is low. As shown In Figure 12b, the F1-score ranged from 64% to 79% for the Shallow CNN and from 68% to 77% for the Tri-CCNN. The Classic LeNet-5 model followed a similar trend in precision, with the spectrogram yellow ROI dataset achieving a sensitivity of 57.17% and an F1-score of 59%, whereas the remaining datasets either reached 100% or 0%. As shown in Figure 13, the confusion matrix revealed that the prediction outcomes of all models were confined to either positive or negative for all tested data, thereby compromising the model’s validation.

Although the Tri-CCNN achieved promising accuracy, the sensitivity (53.43–64.73%) and F1-score (68–77%) were noticeably lower. This discrepancy is largely attributable to the limited dataset size and the imbalance between the control and post-stroke samples, which restricts the model’s ability to learn discriminative patterns for the impaired class. The faint amplitude and frequency features in post-stroke EMG further reduce feature separability, leading to a higher risk of missed detections. Based on the evaluation metrics, the Tri-CCNN model exhibited the promising accuracy and precision across all datasets. The findings also highlight the superior usability of the spectrogram yellow ROI dataset, which achieved optimal accuracy for all CNN models during the training and testing phases. This dataset is particularly valuable because of its ability to provide a comprehensive evaluation of performance metrics, including precision, sensitivity, and F1-score. Notably, this was the only dataset that did not produce a binary outcome in the classification process when the Classic LeNet-5 model was used.

## 4. Discussion

In this study, the architecture of a CNN was systematically examined using EMG recordings in the time–frequency domain (TFR) to differentiate between the characteristics of post-stroke patients and control subjects [70]. To ensure meaningful benchmarking, the proposed Tri-CCNN was evaluated against two established architectures Shallow CNN and LeNet-5 under identical preprocessing, training, and evaluation conditions. This controlled setup enables direct comparison of classification performance independent of data pipeline variations. While prior studies have reported accuracies for classifiers such as SVM, Random Forest [71], CNN [72], LSTM, and hybrid CNN–LSTM [73] models, these results were based on different datasets, signal modalities, or evaluation criteria. Therefore, rather than reproducing those models, we compare our accuracy ranges to published findings to contextualize performance. The observed improvement of Tri-CCNN over both baseline CNNs within the same experimental conditions indicates that the proposed architectural refinements enhance feature extraction from EMG spectrograms, supporting its suitability for stroke-related EMG classification tasks.

The primary objective of this study was to assess the performance of the developed model in comparison with other relevant CNN-based deep learning models and to evaluate their accuracy and effectiveness in distinguishing post-stroke patients from control subjects. The derived 2D dataset spectrogram, Yellow ROI, amplitude spectrum, and peak strength were tested during the classification stage to identify the domains that exhibited more distinctive characteristics for differentiating stroke patients from control subjects.

This study aims to develop an image dataset in 2D format to enhance the analytical potential of EMG signals, thereby providing a more concise and cleaner representation for motor system analysis. However, variations in the signal amplitude and morphology among different subjects can significantly affect the accuracy of EMG signal classification and recognition. Furthermore, the inherent non-linearity and non-stationarity of EMG signals, characterized by changes in amplitude and frequency over time, pose challenges to the generalization capabilities of the model. Although EMG is commonly used for individual case identification [70], its application in predicting and classifying conditions such as stroke requires effective generalization across diverse data categories. This aspect is particularly crucial for assessing the progress of recovery, such as motor system restoration, in stroke patients. To address these challenges, this study proposes utilizing visual representations of EMG signals as a potential strategy to improve the classification accuracy and enhance model generalization.

Post-stroke neuromuscular impairment is commonly reflected in surface electromyography (sEMG) signals through reduced activation amplitude, altered motor unit recruitment, prolonged or fragmented muscle activation, and abnormal frequency characteristics arising from impaired corticospinal control and muscle weakness. Studies have consistently reported decreased EMG power, increased signal variability, delayed onset of activation, and abnormal co-activation patterns in stroke-affected muscles compared with healthy controls, particularly during voluntary motor tasks [74,75,76]. These physiological alterations limit force modulation and coordination and are strongly associated with motor dysfunction severity and recovery potential [77,78]. Time–frequency analysis is therefore well suited for characterizing post-stroke EMG behavior, as spectrogram representations can simultaneously capture amplitude attenuation and frequency-domain irregularities linked to impaired motor unit firing and disrupted neuromuscular control mechanisms [75,76]. Such representations provide a physiologically meaningful basis for distinguishing stroke-affected muscle activity from healthy patterns in automated classification frameworks.

In addition to reduced amplitude and altered frequency characteristics, the post-stroke EMG signals in this study exhibit visually clustered activation patterns during grasp execution. Such clustering is not typically observed in healthy subjects and may reflect impaired motor control mechanisms following stroke. Previous studies have reported that post-stroke spasticity and abnormal muscle synergies disrupt the smooth temporal organization of muscle activation, leading to intermittent, burst-like EMG activity rather than continuous and smoothly modulated signals. Loss of movement smoothness, increased co-contraction, and involuntary muscle activation are common manifestations of upper-limb spasticity after stroke, which may contribute to the clustered EMG patterns observed in the present dataset. These observations are consistent with prior reviews of EMG-based stroke assessment, which associate abnormal temporal EMG structure with spasticity and impaired neuromuscular coordination rather than voluntary force generation alone [79]. While spasticity was not explicitly quantified in this study, the observed clustering supports the physiological relevance of time–frequency analysis for capturing post-stroke motor impairments.

Building on the neuromuscular characteristics discussed above, the TFR was used to visualize and analyze post-stroke EMG behavior. The TFR was presented as a spectrogram, with STFT calculations providing a two-dimensional representation of signal features enhanced by a more effective color scheme that illustrates the typical characteristics of the EMG dataset as in Figure 9. The spectrogram image highlights the significant differences observed between control subjects and post-stroke patients. A clear contrast in the color intensity levels was evident, reflecting the power/frequency, particularly at frequencies between 0.9 and 1 Hz. The pronounced band observed at 0.9–1 Hz in control subjects corresponds to the voluntary contraction–relaxation rhythm generated during sustained hand-grip tasks. This low-frequency component does not represent motor-unit firing rates but rather the temporal periodicity of the force-modulation pattern produced by coordinated recruitment of motor units during a controlled contraction cycle. Healthy individuals typically generate more consistent and synchronized contraction timing, resulting in stable power within this band. In contrast, post-stroke subjects exhibit impaired neuromuscular coordination and irregular contraction rhythms, leading to reduced or fragmented power in the same region. This interpretation aligns with established EMG physiological principles linking voluntary force modulation, motor-unit synchronization, and low-frequency amplitude patterns [80,81,82].

Based on the spectrogram image identification results, comparisons were made focusing on the amplitude of the EMG signal produced. In addition to the spectrogram derived from each contraction segment of the EMG, a second dataset was established that emphasized the yellow ROI. The study also generated two additional 2-dimensional images by referencing previous research approaches to enhance comparison and maximize EMG feature extraction for classification purposes. The EMG amplitude spectrum was examined to illustrate the signal strength distribution at various frequencies by calculating the Fourier Transform value in the frequency domain [64,65,66,67]. Additionally, the peak strength was assessed using a time or frequency domain approach to evaluate the potential properties of motor unit actions through the peak amplitude power distribution of the EMG signal [68,69]. Four datasets were established and utilized for the classification evaluation, and the results demonstrated the superior performance of the spectrogram image focused on the yellow ROI. This dataset achieved optimal accuracy across all the CNN models during the training and testing phases, with a peak accuracy of 93.33% using the Tri-CCNN model. Furthermore, this dataset consistently outperformed the others in terms of precision, sensitivity, and F1-score metrics.

In addition to identifying the most suitable dataset, this study primarily focuses on optimizing the convolutional neural network (CNN) model for dataset classification. Each CNN architecture is trained using four distinct datasets, with the training data selected using both random and systematic methods. These variations were employed to ensure the validity of the trained data, addressing the inherent challenges posed by the diverse range of muscle abilities observed in stroke patients, which may include no activity or muscle electrical activity without movement. To train the proposed model, namely the Tri-CCNN, it was essential to optimize the parameters of each convolution layer by adjusting the input data generated from the previous layer. The reduction in the input size resulting from the previous convolution layer is accompanied by a reduction in parameters, specifically the kernel and filter sizes, allowing for optimal learning of the area and preservation of the resulting features. The complexity of non-linear data in EMG signals is addressed by incorporating a ReLU layer into each pooled layer, thereby enhancing the network’s ability to handle non-linearity [83]. While the primary hyperparameters remained consistent, a low learning rate was employed to address the slow convergence speed associated with a small batch size. Additionally, a softmax layer is introduced at the end to normalize the output and minimize the computational loss.

To assess the robustness of the Tri-CCNN model, a comparative analysis was performed against the Shallow CNN and LeNet-5 models across all datasets. As shown in Figure 11a, Tri-CCNN consistently demonstrated superior performance, achieving a classification accuracy of 93.33%. The performance gap between the Tri-CCNN and the Shallow CNN was 4.73%, which aligns with the findings of [84], emphasizing the considerable impact of the number of convolutional layers on the CNN performance. However, the same study also highlighted that network training was more significantly influenced by the number of convolutional filters and the amount of training data. In this study, the limited availability of the training and testing data reduced the impact of this discrepancy.

Notably, Tri-CCNN achieved a recognition rate of 86.1% on the peak strength dataset, even with the lowest accuracy, highlighting its superior learning effectiveness compared with traditional CNNs. This outcome demonstrates the practical effectiveness of the Tri-CCNN model in applications, such as classifying post-stroke and control EMG data. In contrast, Classic LeNet-5, despite having three convolutional layers, showed a significantly poorer performance. A potential factor contributing to LeNet-5’s underperformance is the use of a 5 × 5 kernel size in each convolutional layer [58]. The optimization technique applied to the Tri-CCNN convolutional layers effectively minimized the information loss from the learned features, further enhancing its performance compared with the other models evaluated in this study.

This result also surpasses the findings of previous studies that examined stroke classification to distinguish stroke patients from non-stroke patients. Studies such as [32,33,34,36,38,39,40,41] have provided valuable insights into stroke classification accuracy using various classifiers, including SVM, CNN, LSTM, Random Forest, and autoregression. In [32,33,34], Random Forest, SVM classifiers, and Linear Regression achieved accuracies of 90.38%, 82%, and 69.5%, respectively. The CNN implementations in [38,39] achieved accuracy rates of 86.66% and 70%, respectively, outperforming the classifiers. In contrast, the approach proposed in [40], which utilized LSTM-Attention, achieved an accuracy of 91.7%. In addition, ref. [36] proposed a combined model integrating the CNN and LSTM methods, yielding an accuracy of 72.95%, which was the highest among the classifiers evaluated. A noteworthy observation from [36] is that incorporating two-dimensional frequency characteristics produces optimal accuracy, surpassing the one-dimensional approach, which reached only 68.38%. Moreover, this study by [41] emphasized the importance of spectrogram data in evaluating EMG signals using power spectral density (PSD).

The integration of the proposed Tri-CCNN model within the framework of this study represents a significant advancement by combining the most effective findings of previous studies. Although the Tri-CCNN achieved a promising accuracy of 93.33%, the sensitivity and F1-score were comparatively lower and, in some dataset combinations, unstable. This discrepancy is primarily attributed to the small dataset size, class imbalance, and the heterogeneity of EMG patterns among post-stroke subjects. These factors create unstable decision boundaries during training, leading to extreme sensitivity values such as 0% or 100%. Consequently, the current model should be considered a preliminary step rather than a ready-to-deploy clinical system. Compared with previous research [40], which achieved an accuracy of 91.7%, the Tri-CCNN model demonstrated comparable accuracy, with other evaluation metrics approaching 90%. Although limited by dataset size, the robust performance of the model across multiple metrics highlights its potential for broader applications.

The high classification accuracy achieved by the Tri-CCNN model not only supports its robustness but also suggests significant translational potential. In clinical practice, EMG-based classification models can serve as non-invasive tools for remote assessment, especially for patients undergoing home-based stroke rehabilitation. Integrating this model into wearable or portable EMG devices may allow clinicians to monitor neuromuscular function remotely and identify changes in muscle activity patterns that reflect recovery progression or the onset of functional decline. Such insights could support decisions regarding therapy adaptation, escalation, or referrals for in-person evaluations.

Furthermore, although the current study did not include longitudinal tracking, it sets the stage for future research on EMG-based progress monitoring. Repeated classification over time could indicate movement toward a “control-like” EMG profile, signaling positive therapy response. Conversely, persistent or worsening classification as “post-stroke” could alert clinicians to plateaued or regressive outcomes. Table 7 lists several clinical scenarios in which the proposed classifier can be integrated into stroke rehabilitation workflows to enhance remote monitoring and decision-making. These cases illustrate the broader potential of EMG-based classification beyond diagnostic discrimination, particularly for supporting personalized and scalable stroke rehabilitation in home and telemedicine settings.

Although the proposed Tri-CCNN model shows promising classification performance and clinical potential, several important limitations must be addressed to enhance its robustness and real-world applicability. The study was based on a relatively small, cross-sectional dataset, limiting its ability to capture the full heterogeneity of post-stroke motor impairment and preventing longitudinal validation of recovery tracking. The dataset also suffered from class imbalance and variability in EMG signal quality, which contributed to the model’s relatively modest sensitivity and F1-scores despite high accuracy and precision—indicating that the classifier is more likely to correctly identify healthy controls while missing a notable portion of stroke cases. Additionally, the age gap between stroke patients (typically >50 years) and healthy controls (20–40 years) introduces a confounder, as age-related physiological differences may influence EMG characteristics.

The study further focused on EMG signals from a single muscle group and a limited number of contraction trials, restricting generalizability across broader motor tasks. Moreover, clinically relevant neuromuscular impairments were not explicitly quantified in the current experimental design. Future work incorporating clinical spasticity scores (e.g., Modified Ashworth Scale) could further elucidate the relationship between clustered EMG activation patterns and spastic motor behavior. Accordingly, further investigations should prioritize larger and more balanced datasets, age-matched control recruitment, richer multi-muscle and multi-task EMG acquisition, and methodological enhancements such as class-weighted learning, oversampling, EMG-specific augmentation, and advanced feature extraction. Longitudinal validation and integration into real-time wearable or tele-rehabilitation platforms are also essential to assess the model’s clinical utility and its potential impact on decision-making and patient outcomes.

## 5. Conclusions

This study presents several significant contributions, including the successful identification of time–frequency domain (TFR) processing as an effective EMG signal processing method, particularly for analyzing amplitude characteristics to identify abnormalities in conditions such as strokes. The study has also highlighted unique features of the two-dimensional image generated from the spectrogram, which reflects power/frequency in dB/(rad/sample) at a frequency of 0.9–1 Hz, and compared the differences observed between normal and abnormal subjects. This finding underscores the potential for predicting and diagnosing motor abilities in stroke cases. Furthermore, the study refined the CNN architecture, resulting in the development of a Tri-CCNN architecture that achieved an average accuracy rate of 93.33% for the spectrogram dataset, thus outperforming other EMG-based classifiers. Overall, the results should be interpreted as an early demonstration of feasibility. While the Tri-CCNN model shows potential for supporting remote rehabilitation monitoring, further validation with larger, balanced datasets is essential before the system can be reliably used in clinical practice. In conclusion, the Tri-CCNN model offers a reliable method for EMG-based classification of post-stroke and healthy individuals. In addition to its technical accuracy, this approach has promising applications in rehabilitation monitoring, remote assessment, and personalized therapy. Future studies should focus on longitudinal datasets to evaluate their role in tracking recovery trajectories and supporting adaptive stroke rehabilitation strategies.

## Figures and Tables

**Table 1:** Data Acquisition Protocol.

Room temperature	25–28 °C
Patient’s position	Supine (lie down)
Skin preparation	Cleaned with alcohol swab
Electrode’s placement	Three in total, include: ✓ 2 electrodes parallel at flexor digitorum profundus muscle belly ✓ 1 electrode in at the elbow as ground
Movement	Grasp for 6 s
Relaxation period	15 s
Data recorded	5 cycles of grasp followed by relaxation

**Table 2:** Segmented Data Format.

Segment	Subject	
	Control (XX)	Patient (YY)
Segment 1	H0XX_01	P0YY_01
Segment 2	H0XX_02	P0YY_02
Segment 3	H0XX_03	P0YY_03
Segment 4	H0XX_04	P0YY_04
Segment 5	H0XX_05	P0YY_05

**Table 3:** The Deep-Learning Hyperparameter Settings for Training the Dataset.

Hyperparameters	Values
Optimizers	SGDM
Learning Rate	0.0001
Mini Batch Size	4
Epochs	10
Loss function	Cross-Entropy
Activation functions	ReLu, softmax

**Table 4:** Summary of Proposed Architecture.

Level	Name	Convolution Kernel Size	Num of Filter	Num of Channel
Input	Input layer			
Layer1	Convolutional layer	5 * 5	8	3
Layer2	MaxPooling2D			
Layer3	ReLuLayer			
Layer4	Convolutional layer	3 * 3	8	4
Layer5	Batch Normalization			
Layer6	MaxPooling2D			
Layer7	ReLuLayer			
Layer8	Convolutional layer	2 * 2	4	2
Layer9	MaxPooling2D			
Layer10	ReLuLayer			
Layer11	Fully Connected			
Layer 12	Softmax			
Output	Output layer			

**Table 5:** Subject Demographic and Health Data Distribution.

	Post-Stroke Patients (*n* = 10)	Healthy Controls (*n* = 10)
**Age** **on admission (years)**		
Mean (SD)	54 (10.93)	30 (8.65)
Range	37–71	21–50
**Gender**		
Male	5 (50%)	5 (50%)
Female	5 (50%)	5 (50%)
**BMI (Kg/m^2^)**		
Underweight (<18.5)	0 (0%)	1 (10%)
Normal weight (18.5–24.9)	7 (70%)	4 (40%)
Overweight (25.0–29.9)	3 (30%)	3 (30%)
Obese (≥30)	0 (0%)	2 (20%)
**Smoking**	3 (30%)	1 (10%)
**Family history of stroke**	1 (10%)	0 (0%)
**Medical history**		
Hypertension	8 (80%)	0 (0%)
Diabetes mellitus	3 (30%)	0 (0%)
Hypercholesterolemia	7 (70%)	1 (10%)
**Blood pressure (mmHg)**		
Mean SBP (SD)	139 (16.67)	127 (16.95)
Mean DBP (SD)	85 (8.16)	81 (10.35)
**MMT**		
0	2 (20%)	0 (0%)
1	0 (0%)	0 (0%)
2	4 (40%)	0 (0%)
3	2 (20%)	0 (0%)
4	2 (20%)	0 (0%)
5	0 (0%)	10 (100%)

**Table 6:** Comparison of Tri-CCNN with Other Tested EMG Classification Models.

Model	Input Feature Type	Conv Layers	Kernel Sizes	Acc (%)	Notes
Tri-CCNN (Proposed method)	Spectrogram (Yellow ROI)	3	5 × 5 → 3 × 3 → 2 × 2	93.33	Optimized for EMG; reduced kernel size to retain locality
Shallow CNN	Spectrogram (Yellow ROI)	1	5 × 5	87.2%	Simple CNN; limited generalization
LeNet-5	Spectrogram (Yellow ROI)	3	5 × 5 → 5 × 5 → 5 × 5	68.33%	Classical architecture; not domain-tuned

**Table 7:** Clinical Application Scenarios.

Application Scenario	Purpose	Model Output Use
**Remote** **Rehabilitation Monitoring**	Track changes in EMG classification over time to infer recovery status	Observe transitions toward control-like EMG patterns
**Home-Based Screening**	Classify stroke severity early using simple motor tasks and EMG sensors	Support diagnosis or triage in low-access areas
**Therapy Adaptation**	Tailor rehabilitation protocols based on neuromuscular feedback	Guide intensity/frequency adjustments based on classification results
**Relapse Detection**	Detect EMG pattern regressions that may indicate stroke recurrence	Trigger alerts for follow-up or urgent evaluation

## Data Availability

The author exclusively provides the datasets created in the present study, which are available upon request. However, it should be noted that the provision of raw data is not possible, due to ethical considerations.
